# Quality Improvement Analyses Revealed a Hidden Shift Following a Retrospective Study on Breastfeeding Rates

**DOI:** 10.1097/pq9.0000000000000347

**Published:** 2020-09-25

**Authors:** Jennifer Sedler, I. Sheevaun Khaki, Carrie A. Phillipi, Dmitry Dukhovny, Kenneth DeVane, Ladawna Gievers

**Affiliations:** From the * Department of Pediatrics, Stanford University, Palo Alto, Calif.; † Department of Pediatrics, Oregon Health and Science University, Portland, Ore.; ‡ School of Medicine, Oregon Health and Science University, Portland, Ore.

## Abstract

Factors affecting exclusive breastfeeding rates are complex. Evaluations for early-onset sepsis can negatively impact breastfeeding success. We sought to determine whether implementing an algorithm utilizing the sepsis risk score (SRS) in chorioamnionitis-exposed newborns would increase exclusive breastfeeding rates. We collaborated with healthcare systems experts to analyze and understand our outcomes.

**Methods:**

We describe a retrospective cohort study of chorioamnionitis-exposed newborns 35 weeks and older gestation in the Mother-Baby Unit at our institution following a quality improvement project that implemented an SRS algorithm. We compared exclusive breastfeeding rates over 2 time periods, 33 months before and 15 months after SRS algorithm implementation. We completed bivariate comparisons using chi-square and Mann–Whitney U tests to understand the factors contributing to exclusive breastfeeding rates. In a secondary analysis, breastfeeding rates and demographic patterns were examined using p-charts.

**Results:**

Following algorithm implementation, exclusive breastfeeding rates increased from 49% to 58% (*P* = 0.10) in chorioamnionitis-exposed newborns. Factors associated with increased exclusive breastfeeding included Caucasian race, English as the primary language, private insurance, vaginal delivery, and positive group B *Streptococcus* status. In the secondary analysis, the proportion of non-Hispanic mothers increased from 63% to 80% during the study.

**Conclusions:**

Despite SRS implementation, exclusive breastfeeding rates increased but not significantly, and certain sociodemographic factors remain associated with exclusive breastfeeding. Secondary analysis revealed an overall demographic shift affecting the dataset, highlighting the importance of thorough data analysis when evaluating a quality improvement project.

## Abstract 2

Supplemental Digital Content is available in the text.

## INTRODUCTION

Breastfeeding is the ideal nutritional source for newborns as it confers multiple benefits for both maternal and infant health.^1,2^ Despite the health benefits, barriers to breastfeeding initiation are multifactorial and can be challenging to address. During the initial newborn hospitalization, reported barriers include maternal socioeconomic status, variable maternal support and education, cultural expectations, and maternal-newborn separation.^3–5^ This study investigated breastfeeding practices following an institutional standard of care practice change utilizing the sepsis risk score (SRS) to improve the management of newborns exposed to chorioamnionitis.^6–8^

Maternal-newborn separation often occurs when a newborn needs a higher level of care, including a concern for early-onset sepsis (EOS). EOS evaluations are one of the most common medical interventions performed on newborns and have been shown to delay the initiation of breastfeeding and reduce exclusive breastfeeding.^3^ Due to the significant morbidity and mortality that may arise from EOS, the Centers for Disease Control and Prevention (CDC) previously recommended early, and empiric, evaluation of all newborns with risk factors for EOS, including a maternal diagnosis of chorioamnionitis.^9^ Although these recommendations were designed to identify and empirically treat newborns at risk for EOS, there was a substantial risk for overtreatment which could, in turn, increase maternal-newborn separation and impact breastfeeding success.

Recently, a risk-based calculator was developed to better stratify and treat newborns at the highest risk of developing EOS based on the determination of the SRS.^6,7^ Management strategies utilizing the SRS have been shown to significantly decrease sepsis evaluations and maternal-newborn separation in managed care organizations and academic centers, including at our institution.^8,10^ At Oregon Health and Science University (OHSU), for chorioamnionitis-exposed newborns, the use of the SRS led to a decrease in neonatal intensive care unit (NICU) observations from 72.8% to 9.8%, antibiotic exposure from 95.3% to 9.1%, and laboratory evaluations from 95.8% to 21.7%, when compared to the CDC guidelines.^8^ The most recent CDC guidelines allow for management decisions that utilize the SRS.^11^ We hypothesized that by decreasing the maternal-newborn separation of chorioamnionitis-exposed newborns, exclusive breastfeeding rates would increase. Our study’s objectives were to (1) compare exclusive breastfeeding rates before and after routine use of the SRS for all well-appearing chorioamnionitis-exposed newborns and (2) identify factors attributable to exclusive breastfeeding success in this patient population.

Additionally, although we will present the medical findings of our study in this report, we intend to highlight a discussion surrounding how new results can be uncovered by collaborating with healthcare systems experts. Like many professional medical centers across the nation, OHSU shares the mission to continually improve the quality of care provided to its patients by utilizing quality improvement (QI) science. OHSU has structurally solidified this QI initiative by collaborating with healthcare systems experts. Our research team partnered with a healthcare systems engineer to critically analyze our current research project and its findings.

## METHODS

### Design

**Fig. 1.**
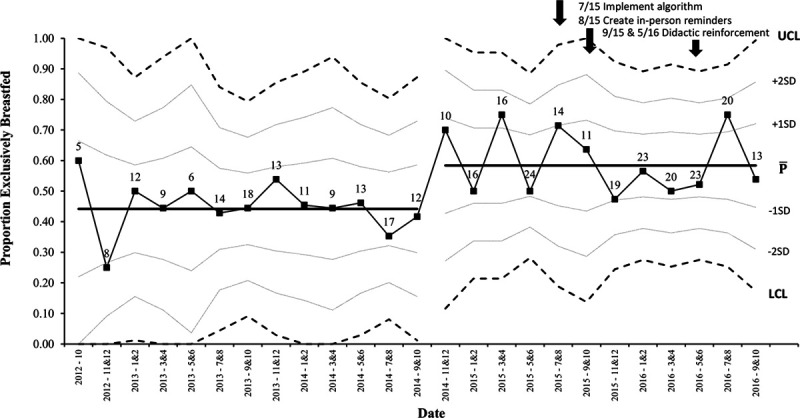
P-chart demonstrating the bimonthly proportion of chorioamnionitis-exposed neonates who exclusively breastfed over the study period. LCL indicates lower control limit; UCL, upper control limit.

The parent phase of this study was a QI project to develop an algorithm for implementing the SRS in the management of chorioamnionitis-exposed newborns older than 35 weeks’ gestation admitted to the mother-baby unit (MBU) at OHSU (**see Figure 1, Supplemental Digital Content 1,** which describes OHSU workflow algorithm for newborns exposed to chorioamnionitis utilizing the sepsis risk score and newborn clinical examination, http://links.lww.com/PQ9/A210). The SMART aim was to reduce (1) antibiotic exposure by 50%; (2) laboratory evaluations by 40%; and (3) maternal-neonatal separation by 50% from baseline by 1 year following algorithm implementation. Key stakeholders, including pediatric and family medicine providers and nurses from the MBU, NICU, and labor and delivery ward, were identified and assembled into a multidisciplinary working group. After reviewing the literature, an algorithm incorporating the SRS was developed to promote individualized evaluation and treatment.^8^ The algorithm was revised based on feedback from the multidisciplinary group and incorporated into the electronic medical record (EMR). Plan-Do-Study-Act methodologies were used for implementation and to assess the subsequent change. These changes included electronic and in-person reminders, along with didactic educational sessions to reinforce the algorithm. The QI team met monthly to measure outcomes, formally review algorithm feedback, review algorithm compliance, and to assess balancing measures, including missed cases of EOS and readmission rates.

The current, retrospective second phase of this project evaluated the impact of the SRS algorithm on breastfeeding rates for chorioamnionitis-exposed newborns. Our primary outcome was exclusive breastfeeding rates at hospital discharge, compared over 2 time periods: (1) 33 months during which management decisions were based on the CDC guidelines and (2) 15 months after SRS implementation in July 2015. The OHSU Institutional Review Board reviewed and approved this study.

### Setting

OHSU is a quaternary care academic medical center in Portland, Oregon, with approximately 2,500 live births per year. Formula and human donor milk (HDM) are available as breastmilk substitutes for newborns at OHSU. Only mothers’ milk (direct or expressed with or without fortification) is considered exclusive breastfeeding in this study. Indications for supplementation did not change throughout the study. During the study period, OHSU introduced HDM (October 2013) as an option for supplementation. The most recent CDC breastfeeding report card for Oregon (2018) indicates an 89.4% rate of ever-breastfed compared to a US national average of 83.2%, and 33.4% exclusive breastfeeding at 6 months compared to 24.9% nationally.^12^

At our institution, before July 2015, chorioamnionitis-exposed newborns were managed according to the 2010 CDC guidelines for secondary prevention of early-onset group B *Streptococcus* (GBS) disease.^8^ The standard of care was to observe these infants in the NICU for at least 4 hours before transfer to the MBU, obtain a blood culture, and administer empiric antibiotics for 48 hours. As of July 2015, evaluation and management of these newborns incorporated the SRS and avoided routine NICU observation, laboratory evaluation, and empiric antibiotics unless indicated by the SRS.

### Sample

The sample population consisted of all maternal-newborn dyads who received a clinical diagnosis of chorioamnionitis during the study period and diagnosed at the delivery provider’s discretion. Inclusion criteria for newborns included birth at OHSU and gestational age (GA) at birth older than 35 weeks. Exclusion criteria included major birth defects as defined by the Vermont Oxford Network, admission to the NICU for >8 hours, or gestational age younger than 35 weeks due to obligatory admission to the NICU per OHSU policy. We defined supplementation as the receipt of either formula or HDM.

### Data Collection

The EMR was queried by a study team member (J.L.S.) to identify women who developed an intrapartum fever (*>*100.4°F); maternal and newborn charts were reviewed to confirm documentation of a clinical diagnosis of chorioamnionitis. Maternal clinical and sociodemographic factors were collected from the EMR, including maternal race, ethnicity and primary language, maximum maternal temperature, GBS status, and insurance type. Newborn data included birth weight, gestational age at birth, gender, mode of delivery, SRS utilization, antibiotic exposure, time spent in the NICU, and receipt of supplemental feeds.

### Data Analysis

Exclusive breastfeeding rates were compared using chi-square tests and plotted on a p-chart. Chi-square test, tests of binomial proportions, ANOVA, and Mann–Whitney U tests were used to assess continuous and categorical sociodemographic variables. Univariate and stepwise multivariable logistic regression analyses were performed to determine which variables were associated with exclusive breastfeeding. In partnership with a healthcare system engineer, these variables were plotted on p-charts to identify special cause variation. A post-hoc analysis was performed to understand if the chorioamnionitis-exposed cohort was different from the overall MBU population. We collaborated with 2 study team members with expertise in performance improvement statistics, including a healthcare systems engineer, to perform this analysis. They performed the analyses using SPSS Version 25 (IBM Corp, Armonk, N.Y.).

## RESULTS

Three hundred and fifty-six newborns met inclusion criteria; 213 chorioamnionitis-exposed dyads were identified before the implementation of SRS application and 143 dyads after. Significant demographic differences between the patient populations included an older mean maternal age and a greater proportion of Caucasian and non-Hispanic mothers in the postimplementation cohort (Table 1). Exclusive breastfeeding rates increased (49.1%–57.8%, *P = 0*.10) following SRS implementation. Factors significantly associated with increased exclusive breastfeeding rates among chorioamnionitis-exposed newborns included Caucasian race, non-Hispanic ethnicity, English as the primary language, private insurance, vaginal delivery, and GBS positive status (Table 2). Fifty-three percent of well-appearing newborns born to mothers with chorioamnionitis were exclusively breastfeeding at hospital discharge compared to 68% of well-appearing unexposed newborns (*P = 0*.05).

**TABLE 1.** Maternal and Neonatal Demographics and Clinical Characteristics

	Prealgorithm, n = 213	Postalgorithm, N = 143	*P*
Age (years), mean ± SD	28.7 ± 5.7	30.3 ± 6.4	<0.05
C-section delivery, n (%)	87 (40.8)	57 (39.9)	0.94
Intrapartum maximum temperature (°C), mean ± SD	38.5 ± 0.5	38.5 ± 0.5	0.28
GBS status, positive, n (%)	56 (26.3)	34 (23.8)	0.42
Race, n (%)			<0.05
Caucasian	115 (54.0)	94 (65.7)	
Multiracial	66 (31.0)	19 (13.3)	
Asian	14 (6.5)	12 (8.4)	
Black	6 (2.8)	4 (2.8)	
Other	3 (1.4)	0	
Unknown/decline	9 (4.2)	14 (9.8)	
Ethnicity, n (%)			0.01
Non-Hispanic	149 (70.0)	111 (77.6)	
Hispanic	56 (26.2)	20 (14.0)	
Unknown	8 (3.8)	12 (8.4)	
Language, non-English, n (%)	35 (16.4)	14 (9.8)	0.10
Insurance, public, n (%)	112 (52)*	62 (43)†	0.10
Infant characteristics			
Male gender, n (%)	108 (50.7)	84 (58.7)	0.16
Gestational age at birth (weeks), mean ± SD	39.9 ± 1.3	39.8 ± 1.3	0.44
Birth weight (g), mean ± SD	3538 ± 477	3526 ± 479	0.82
Apgar score, 5 mins, mean ± SD	8.6 ± 0.9	8.6 ± 0.9	0.55
Exclusive breastfeeding, n (%)	104 (49.1)	83 (57.8)	0.10

*Other insurance types included private (n = 96), military (n = 3), and self-pay (n = 2).

†Other insurance types included private (n = 79) and military (n = 2). GBS, group B *Streptococcus*.

**TABLE 2.** Comparing Maternal and Newborn Characteristics between Chorioamnionitis-exposed Patients Who Were Exclusively Breastfed and Those Who Were Supplemented

	Patients Who Were Exclusively Breastfed, n = 187	Patients Who Were Supplemented, n = 169	***P***
Maternal characteristics			
Age (years), mean ± SD	30.1 ± 5.6	30.0 ± 6.8	0.99
Vaginal delivery, n (%)	131 (70)	81 (48)	<0.001
Intrapartum maximum temperature	38.5 ± 0.4	38.5 ± 0.5	0.99
(°C), mean ± SD			
GBS status, positive, n (%)	59 (32)	33 (20)	0.01
Race, n (%)			<0.001
Caucasian	127 (68)	83 (49)	
Multiracial	25 (13)	60 (36)	
Asian	11 (6)	15 (9)	
Black	6 (3)	5 (3)	
Other	1 (1)	2 (1)	
Unknown/decline	17 (9)	4 (2)	
Ethnicity, n (%)			<0.001
Non-Hispanic	144 (77)	116 (69)	
Hispanic	27 (14)	49 (29)	
Unknown	16 (9)	4 (2)	
Language, non-English, n (%)	12 (6)	37 (22)	<0.001
Insurance*, Public, n (%)	72 (38)	101 (60)	<0.001
Infant characteristics			
Female gender, n (%)	87 (47)	77 (46)	0.77
Gestational age at birth (wks)	39.7 ± 1.2	39.6 ± 1.3	0.45
Mean ± SD			
Birth weight (g), mean ± SD	3534 ± 423	3510 ± 531	0.99
Antibiotic exposure, n (%)	108 (58)	108 (64)	0.28

*This analysis was run using n = 348 patients. Those with military insurance and self-pay were excluded. GBS, group B Streptococcus.

**Fig. 2.**
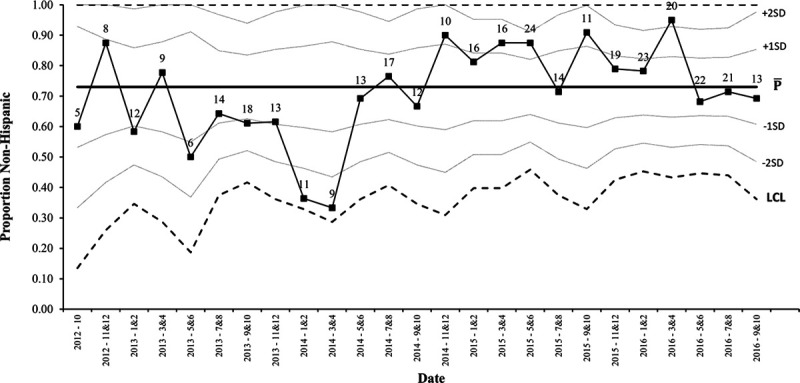
P-chart demonstrating the bimonthly proportion of chorioamnionitis-exposed mothers who were non-Hispanic over the study period. LCL indicates lower control limit; UCL, upper control limit.

An upward shift in the process stage mean of exclusive breastfeeding rates increased before SRS implementation (Fig. 1).^13^ Comparative statistics revealed a significant change in the racial and ethnic demographics of prealgorithm and postalgorithm implementation cohorts. Univariate analysis identified 6 potentially influential sociodemographic variables, including delivery method, language, race, ethnicity, GBS status, and insurance type. These variables were subsequently plotted on p-charts to analyze whether they shifted concurrently with the upward shift in exclusive breastfeeding. Signals of special cause variation were present for race and ethnicity, specifically of non-Hispanic patients (Fig. 2). There was an upward shift in the proportion of non-Hispanic patients. Analyses of all dyads admitted to the MBU revealed that this shift in demographics was present throughout the MBU population, as the number of non-Hispanic patients increased from 74% to 80%, utilizing the shift point of October 2014. The proportion of non-Hispanic mothers who exclusively breastfed did not increase over time, whereas the percentage of exclusively breastfeeding Hispanic mothers increased (**see Figure 2a and b, Supplemental Digital Content 2,** which describes (a) P-chart demonstrating the bimonthly proportion of non-Hispanic chorioamnionitis-exposed mothers who exclusively breastfed over the study period. The first vertical dashed line represents the time (October 2014) of the demographic shift (increase in the proportion of non-Hispanic patients). The second vertical dashed line represents the timing of the hospital algorithm implementation (July 2015). (b) P-chart demonstrating the quarterly proportion of Hispanic chorioamnionitis-exposed mothers who exclusively breastfed over the study period. The first vertical dashed line represents the time (October 2014) of the demographic shift (increase in the proportion of non-Hispanic patients). The second vertical dashed line represents the timing of the hospital algorithm implementation (July 2015), http://links.lww.com/PQ9/A210). Multivariable analysis demonstrated 6 significant variables associated with exclusive breastfeeding, including gestational age, delivery method, language, race, GBS status, and time in NICU (Table 3). Insurance status and ethnicity were no longer significant in the multivariable analysis. However, gestational age and time in NICU were significant. The implementation of the SRS was not associated with exclusive breastfeeding in a multivariable model.

**Table 3.** Multivariable Analysis Demonstrating Significant Variables Associated with Exclusive Breastfeeding

Variable	Odds Ratio (95% CI)	Interpretation
Gestational age at birth, wks	0.70 (0.57–0.84)	Less likely to supplement with increasing gestational age
Delivery method, cesarean	3.14 (1.90–5.16)	More likely to supplement with cesarean delivery
Language, Spanish	2.50 (0.96–6.50)	More likely to supplement if Spanish-speaking
Language, other*	8.56 (2.18–33.58)	More likely to supplement if non-English and non-Spanish-speaking
Race, multiracial	3.13 (1.71–5.73)	More likely to supplement if multiracial
GBS status, positive	0.56 (0.32–0.98)	Less likely to supplement if GBS positive
Time in NICU, h	1.12 (1.04–1.33)	More likely to supplement with more time in NICU

*This analysis includes languages other than English and Spanish. CI, confidence interval; GBS, group B *Streptococcus*; NICU, neonatal intensive care unit.

## DISCUSSION

In the setting of a recent practice change that successfully reduced maternal-newborn separation, exclusive breastfeeding rates increased, though not reaching statistical significance. The decision to provide supplemental feedings is complex, nuanced, and requires shared-decision making. There are no clear indications to initiate supplementation outside of medical contraindications to breastfeeding at our institution. We speculate that the chorioamnionitis-exposed newborns within the study cohort were perceived differently in terms of their disease susceptibility, which may have driven the decision to supplement. There is evidence that vulnerability factors, such as exposure to second-hand smoke, postpartum depression, and lower levels of maternal education, are risk factors for diminished exclusive breastfeeding rates.^14^ Reassuringly, educating mothers and providing reassurance have been shown to impact maternal confidence and willingness to breastfeed positively.^14^ Despite efforts made at our institution to individualize each newborn’s risk of developing EOS and managing them accordingly, they may still be viewed through the lens of impending illness and thus fall short of the exclusive breastfeeding rates of their counterparts. Providers may have a lower threshold for offering supplementation and may be less comfortable providing reassurance to families given the higher sepsis risk.

Further investigation within this dataset confirmed similar findings to the literature of common factors that may affect exclusive breastfeeding rates, including maternal race, primary language, insurance, mode of delivery, and GBS status.^15,16^ Studies have shown that women in certain racial minority groups who live in areas with less access to maternity care that support breastfeeding are less likely to breastfeed exclusively.^17^ Additionally, although Hispanic women are more likely to initiate breastfeeding, acculturation has negatively impacted exclusive breastfeeding initiation rates in the United States.^18–21^ Hispanic women have also cited various barriers such as discouragement after not achieving early success, modesty or embarrassment, work-related restrictions, the uncertainty of milk supply, and lack of support from family or partners.^22–27^ Language barriers and cultural heterogeneity among patients and the healthcare system may make breastfeeding education more challenging.

The process of reflecting on research methodologies revealed specific limitations in this study. Utilizing a QI framework (specific aims, Plan-Do-Study-Act cycles), the SRS calculator was successfully implemented to care for chorioamnionitis-exposed newborns.^8^ Following this initial, parent project, which decreased maternal-newborn separation, exclusive breastfeeding rates became a necessary outcome to evaluate. Comparative statistics evaluated the impact of this change on breastfeeding success. However, we discovered an important hidden shift in the demographics by analyzing the data using p-charts. Specifically, comparative statistics demonstrated a significant difference in the ethnicity of the cohort prealgorithm and postalgorithm implementation. However, after collaborating with a healthcare systems expert, special cause variation was noted in the proportion of non-Hispanics before the study intervention. Early involvement of a healthcare systems expert likely would have prevented the erroneous conclusion as additional statistical methods would have discovered the shifting demographics.

The transition between QI and comparative statistics is both a strength and limitation of this study. The strength lies within the ability of different types of statistical analyses to highlight unique findings. However, using 2 statistical methods limits the reproducibility of this study. A second possible limitation is the duration of the study. While a nonstatistically significant increase in breastfeeding rates occurred during the study, if the study duration was extended, results may have differed. One could hypothesize that by intervening less (less antibiotic exposure and laboratory evaluations) on low-risk, chorioamnionitis-exposed newborns, over time, they will be viewed as less vulnerable, which ultimately could improve exclusive breastfeeding rates.

One final limitation is that over the study period, OHSU newly incorporated HDM as an option for supplementation. The effect of these changes on breastfeeding was not studied, given a lack of standardized documentation within the EMR. Although HDM introduction could be considered a potential confounder, exclusive breastfeeding rates remained stable following its introduction in October 2013.

Although the initial interpretation of our data led us to the conclusion that breastfeeding rates improved due to algorithm implementation, further analysis in collaboration with a health care systems engineer, revealed that a shift occurred months prior (Fig. 1). We explored various elements that may have influenced this shift and identified an increase in non-Hispanic patients beginning in October 2014. There was a similar coinciding change in the overall MBU population at that time. Further exploration into our institution’s shifting demographics did not reveal a known cause, but one could speculate impacts from changes in immigration patterns, insurance status, or access to obstetric care. This population-wide change likely contributed to our study findings, given that ethnicity affects breastfeeding rates. Exclusive breastfeeding rates in this study’s non-Hispanic population remained steady over time (mean proportion of 55.4%) and increased in the Hispanic population (24.5%–57.7%, *P* < 0.05). Even though exclusive breastfeeding rates increased in our Hispanic population, due to the small sample size, we suspect that the overall higher proportion of non-Hispanic patients was more impactful for overall breastfeeding rates. Therefore, it may be that the changing demographics affected exclusive breastfeeding rates more substantially than our practice change. Discovering this demographic shift underscored the importance of analyzing data from different angles with a widened expertise to understand the population milieu better.

Our findings highlight that specific data analysis methods can either illuminate or conceal crucial factors affecting outcomes when performing QI work. QI is an important and increasingly popular means of systematically improving the care of individual patients.^28,29^ As health systems evolve to incorporate QI science into their standard operations, clinicians must develop a deeper understanding regarding QI methodologies. Like the American Academy of Pediatrics, many major accrediting bodies are committed to using QI to promote the health and well-being of children and the systems providing their care. As a continued source of momentum, the National Academy of Engineering recently partnered with the Institute of Medicine to highlight the importance of healthcare professionals collaborating with a broader array of disciplines, including healthcare systems engineers, to gain knowledge and techniques for improving the healthcare delivery system.^30,31^

When seeking to improve the quality of health care through QI science, it is imperative to scrutinize the data and understand the patient population. Upon further reflection of our data, we identified that a naïve assumption led to improper initial interpretation of our study results. Specifically, we assumed that breastfeeding rates increased (though not significantly) due to decreased maternal-newborn separation following SRS implementation. However, collaboration with a healthcare system engineer led to the discovery of the demographic changes that were likely more influential on breastfeeding rates. This study underscores the value of dissecting study findings with partners across disciplines to authenticate conclusions. As stewards of both individual and population-based healthcare, we must foster collaborative, interprofessional relationships, as we did with a healthcare system engineer, to promote effective change best.

## DISCLOSURE

Dr. Dukhovny is a consultant for Vermont Oxford Network. The other authors have no financial interest to declare in relation to the content of this article.

## ACKNOWLEDGMENTS

All phases of this study were supported by the Friends of Doernbecher research grant.

Preliminary data for this article was presented at Pediatric Academic Societies.

## Supplementary Material


