# Supplementary material for: Quality Improvement Analyses Revealed a Hidden Shift Following a Retrospective Study on Breastfeeding Rates
Source: Pediatr Qual Saf. 2020 Sep 25;5(5):e347. doi: 10.1097/pq9.0000000000000347 (PMC8487780; doi:10.1097/pq9.0000000000000347)
Supplement: Supplementary file 1 [file pqs-5-e347-s001.pdf]

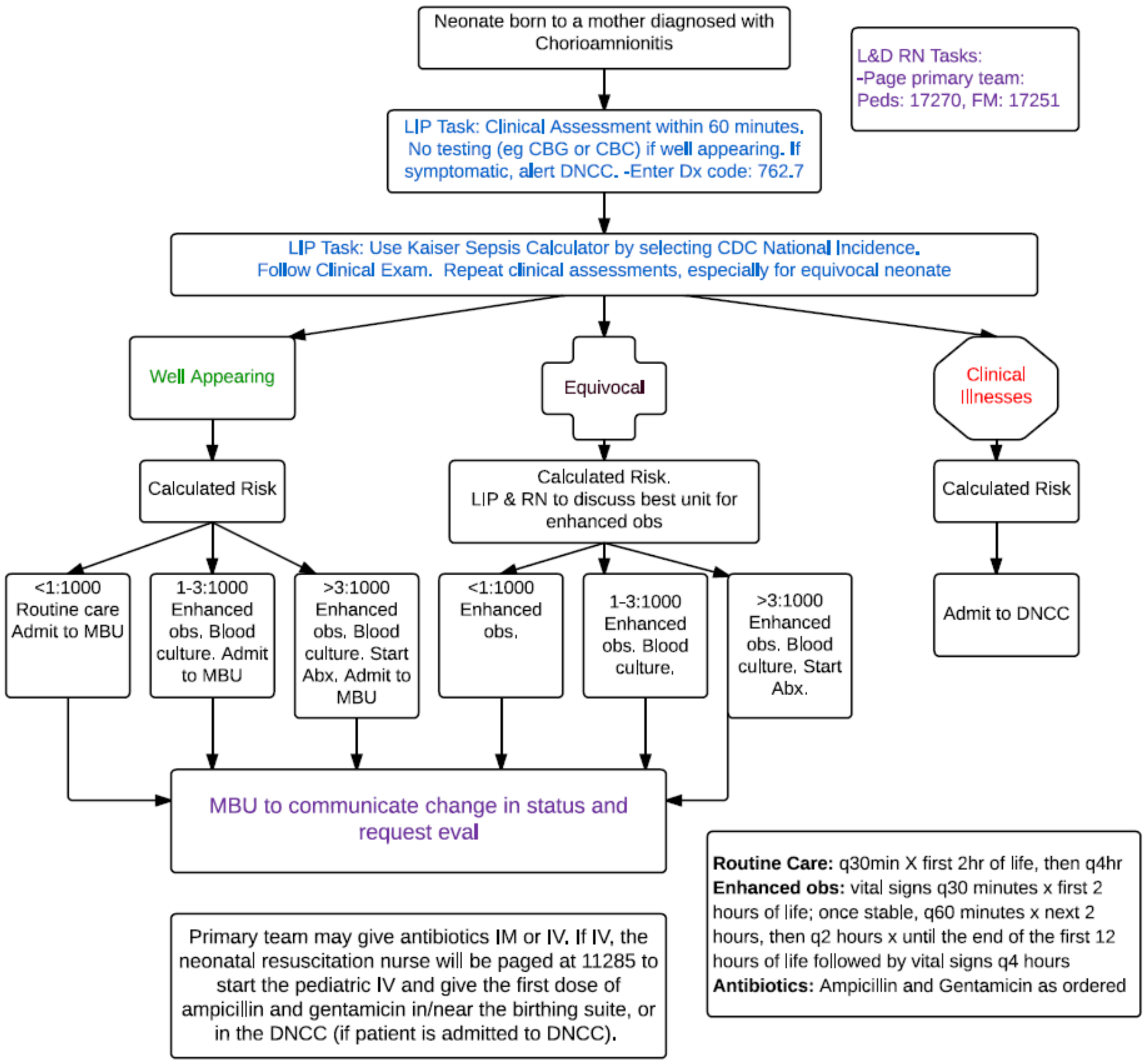

Supplemental Figure 1: OHSU workflow algorithm for newborns exposed to chorioamnionitis

LIP, licensed individual practitioner; CBG, capillary blood glucose; CBC, complete blood count; DNCC, Doernbecher Neonatal Care Center (NICU); Dx, diagnosis; L&D, labor & delivery; FM, family medicine; CDC Centers for Disease Control and Prevention; MBU, mother baby unit; Abx, antibiotics; IM, intramuscular; IV, intravenous

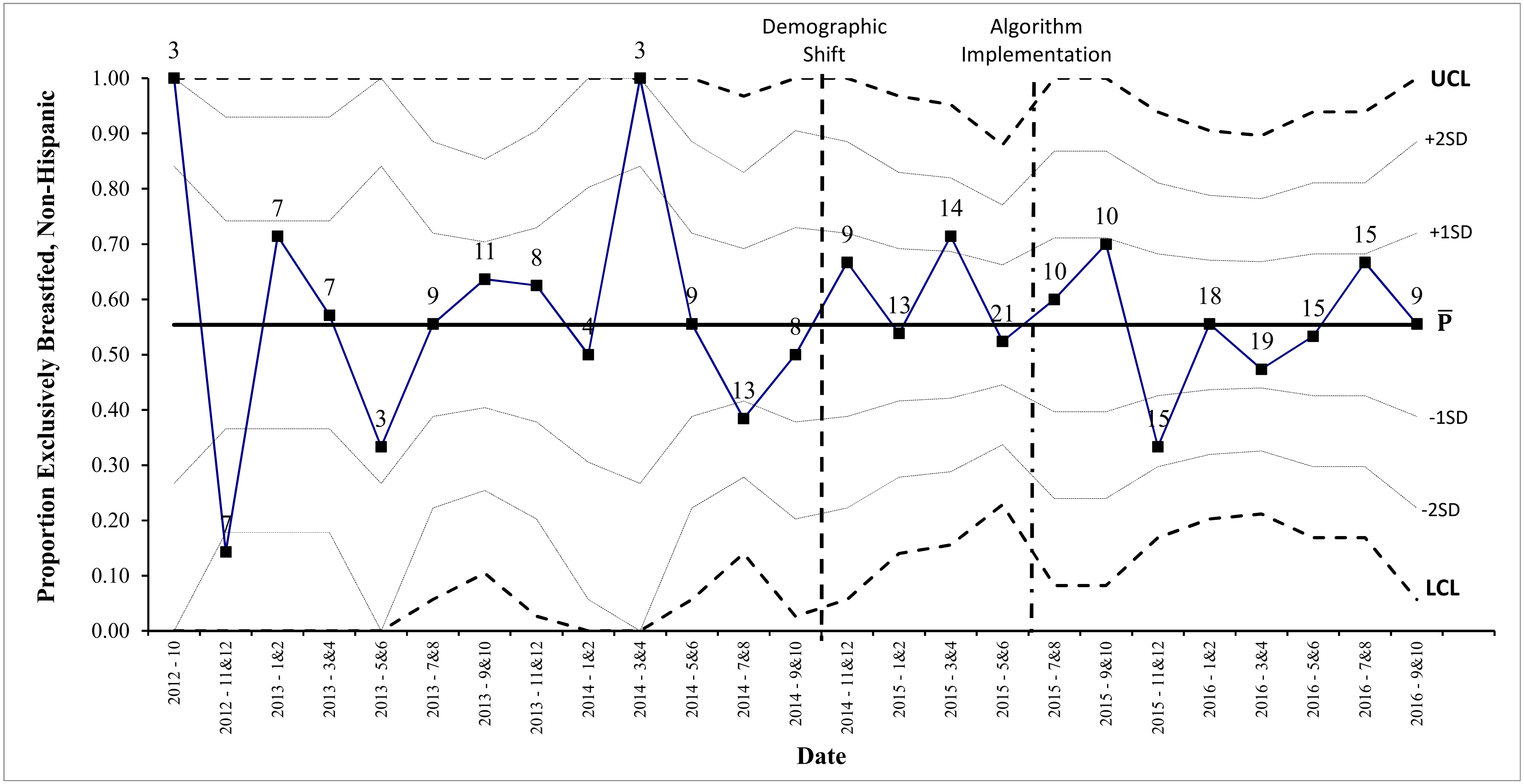

Demographic Shift
Algorithm Implementation
UCL
+2SD
+1SD
P̄
-1SD
-2SD
LCL
Proportion Exclusively Breastfed, Non-Hispanic
1.00
0.90
0.80
0.70
0.60
0.50
0.40
0.30
0.20
0.10
0.00
2012 - 10
2012 - 11&12
2013 - 1&2
2013 - 3&4
2013 - 5&6
2013 - 7&8
2013 - 9&10
2013 - 11&12
2014 - 1&2
2014 - 3&4
2014 - 5&6
2014 - 7&8
2014 - 9&10
2014 - 11&12
2015 - 1&2
2015 - 3&4
2015 - 5&6
2015 - 7&8
2015 - 9&10
2015 - 11&12
2016 - 1&2
2016 - 3&4
2016 - 5&6
2016 - 7&8
2016 - 9&10
Date

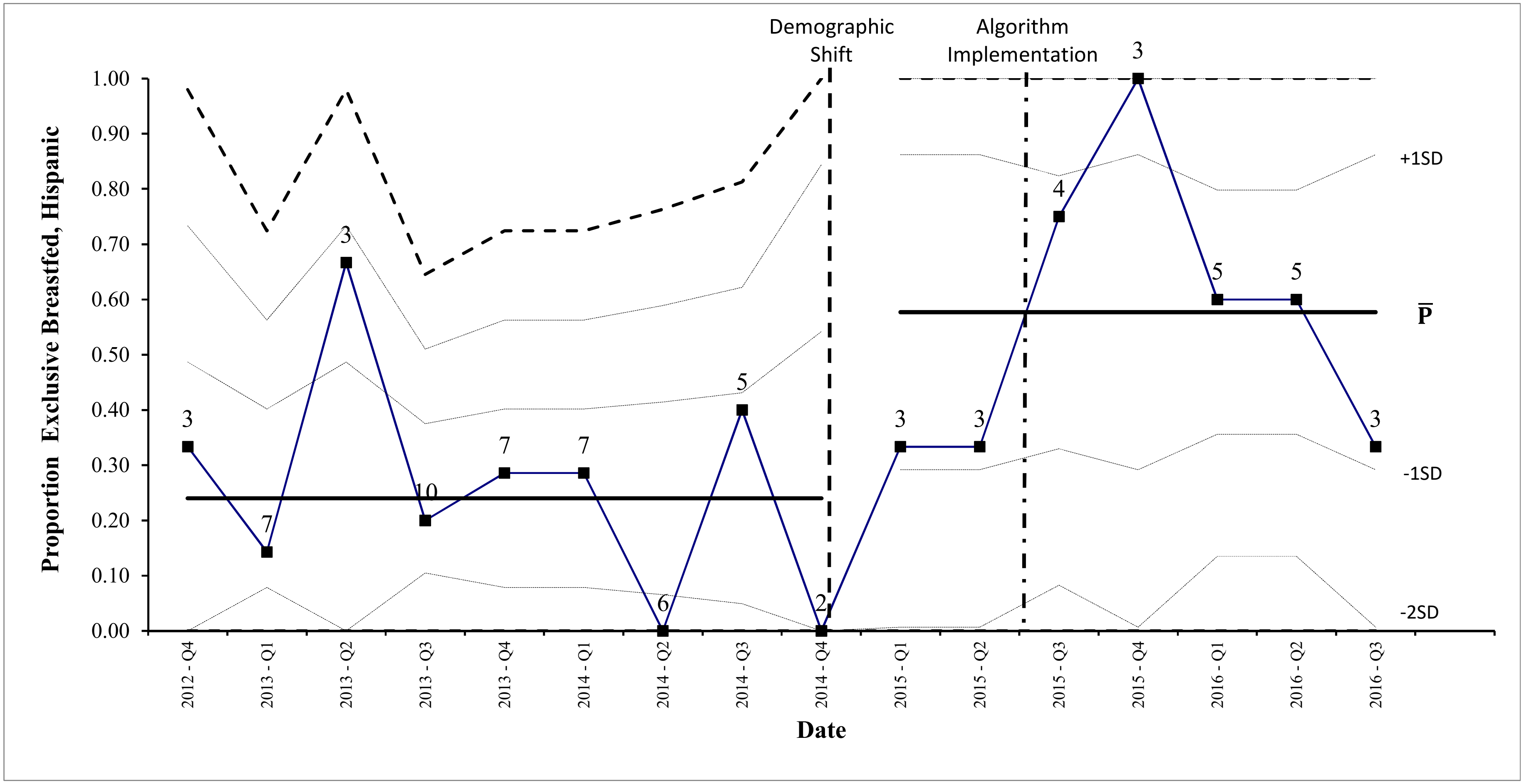

Demographic Shift
Algorithm Implementation
Proportion Exclusive Breastfed, Hispanic
1.00
0.90
0.80
0.70
0.60
0.50
0.40
0.30
0.20
0.10
0.00
3
7
3
10
7
7
6
5
2
3
3
4
3
5
5
3
+1SD
P̄
-1SD
-2SD
2012 - Q4
2013 - Q1
2013 - Q2
2013 - Q3
2013 - Q4
2014 - Q1
2014 - Q2
2014 - Q3
2014 - Q4
2015 - Q1
2015 - Q2
2015 - Q3
2015 - Q4
2016 - Q1
2016 - Q2
2016 - Q3
Date
